# Social network approaches to locating people recently infected with HIV in Odessa, Ukraine

**DOI:** 10.1002/jia2.25330

**Published:** 2019-06-27

**Authors:** Leslie D Williams, Ania Korobchuk, Pavlo Smyrnov, Yana Sazonova, Georgios K Nikolopoulos, Britt Skaathun, Ethan Morgan, John Schneider, Tetyana I Vasylyeva, Yen T Duong, Svitlana Chernyavska, Vitaliy Goncharov, Ludmila Kotlik, Samuel R Friedman

**Affiliations:** ^1^ National Development and Research Institutes New York NY USA; ^2^ Alliance for Public Health Kyiv Ukraine; ^3^ Medical School University of Cyprus Nicosia Cyprus; ^4^ Division of Global Public Health University of California San Diego San Diego CA USA; ^5^ University of Chicago Chicago IL USA; ^6^ Department of Medicine and Center for HIV Elimination University of Chicago Chicago IL USA; ^7^ New College University of Oxford Oxford UK; ^8^ ICAP‐NY Columbia University New York NY USA; ^9^ Odessa Regional Laboratory Center of the Ministry of Health of Ukraine Odessa Ukraine; ^10^ Center for Drug Use and HIV Research New York NY USA; ^11^ Department of Population Health New York University Medical School New York NY USA

**Keywords:** HIV, treatment as prevention, social network, intervention, recent HIV infection, PWID, Ukraine

## Abstract

**Introduction:**

This paper examines the extent to which an intervention succeeded in locating people who had recently become infected with HIV in the context of the large‐scale Ukrainian epidemic. Locating and intervening with people who recently became infected with HIV (people with recent infection, or PwRI) can reduce forward HIV transmission and help PwRI remain healthy.

**Methods:**

The Transmission Reduction Intervention Project (TRIP) recruited recently‐infected and longer‐term infected seeds in Odessa, Ukraine, in 2013 to 2016, and asked them to help recruit their extended risk network members. The proportions of network members who were PwRI were compared between TRIP arms (i.e. networks of recently‐infected seeds vs. networks of longer‐term infected seeds) and to the proportion of participants who were PwRI in an RDS‐based Integrated Biobehavioral Surveillance of people who inject drugs in 2013.

**Results:**

The networks of PwRI seeds and those of longer‐term infected seeds had similar (2%) proportions who were themselves PwRI. This was higher than the 0.25% proportion in IBBS (OR = 7.80; *p* = 0.016). The odds ratio among the subset of participants who injected drugs was 11.17 (*p* = 0.003). Cost comparison analyses using simplified ingredients‐based methods found that TRIP spent no more than US $4513 per PwRI located whereas IBBS spent $11,924.

**Conclusions:**

Further research is needed to confirm these results and improve TRIP further, but our findings suggest that interventions that trace the networks of people who test HIV‐positive are a cost‐effective way to locate PwRI and reduce HIV transmission and should therefore be implemented.

## 1 Introduction

A major part of the global HIV/AIDS prevention strategy focuses on treatment as prevention 1. Finding and intervening with people soon after they get infected is an important part of this strategy because transmission is relatively frequent during the period of early infection 2, 3, 4, 5, 6 due to high viral loads 7, 8, 9, relatively low immune response 10, and (possibly temporary) high rates of risky behaviours 11. The logic for focusing on people with recent infection (PwRI) has been previously described 5, 12. Research from Athens (Greece) provides evidence that recruiting the risk networks of PwRI is a good strategy for finding additional PwRI, since in that study, PwRI were recruited at higher rates in the risk networks of PwRI seeds than in the comparison group risk networks of longer‐term infected seeds 13.

The Transmission Reduction Intervention Project (TRIP) conducted risk network‐based recruiting and HIV counselling and testing in Odessa, Ukraine. It focused on locating PwRI to prevent additional transmissions by them and their network members.

This paper compares the proportions of PwRI in two arms of TRIP: (1) the risk networks of PwRIs and (2) the risk networks of longer‐term‐infected people. It also compares the proportions of PwRI in the TRIP networks with the proportions of PwRI located in an independent project that was conducted among people who inject drugs (PWID) in Odessa's Integrated Biobehavioral Surveillance (IBBS). Finally, since costs can affect the feasibility of interventions, we compare the costs of locating PwRI in TRIP with the costs of locating PwRI using respondent‐driven sampling (RDS) in IBBS.

## 2 Methods

### 2.1 Geographic setting

Eastern Europe and Central Asia are regions with a growing HIV‐epidemic in which the majority of cases are concentrated among people who inject drugs (PWID) and their sexual partners 14, 15. In 2018, Ukraine had an estimated 243,000 people living with HIV (PLHIV), reflecting 0.64% HIV‐prevalence among the adult population 16. The Ukrainian epidemic is primarily concentrated among three key populations: PWID, sex workers (SWs), and men who have sex with men (MSM), with HIV‐prevalence 22.6%, 5.2% and 7.5% respectively. There is some evidence that political upheaval and subsequent war may have reversed the beginnings of a decline in the epidemic 17.

Odessa city is located in southern Ukraine. The estimated 29,500 PLHIV in the Odessa region constitute almost 12% of the national total 18. Official supervision of PLHIV by the AIDS Center provides evidence about HIV indicators in the Odessa region, where 2593 newly detected HIV cases were officially registered in 2018, a rate of 109 cases per 100,000 population 19. HIV in the Odessa region is also concentrated among key populations, with an estimated prevalence of 18.7% among PWID; an estimated prevalence of 13.0% among MSM; and an estimated 3.0% among sex workers 20, 21.

### 2.2 TRIP methods

#### 2.2.1 TRIP eligibility criteria and arms

TRIP was a two‐arm network intervention study in Odessa, Ukraine, from November 2013 to March 2016: (1) An intervention arm comprised of members of extended risk networks of recently infected seeds, and (2) A comparison arm composed of longer‐term HIV‐positive seeds’ extended risk network members of longer‐term positive seeds. Potential “seeds” (initial participants) were people referred to TRIP by AIDS Centers and NGOs because they were thought to be relatively likely to have been recently infected with HIV. Those who met the criteria described below were classified as “recently infected seeds” (N = 24). Those who were not recently infected were classified as “longer‐term infected seeds” (N = 18).

Seeds’ risk network members were eligible for TRIP. We operationalized “risk networks” as people with whom participants injected drugs, people with whom they had sex, people who were present while participants were having sex or using drugs, and people recruited from small‐size “venues” where participants injected drugs or located sex partners. This definition of risk networks was used since it seemed likely to include people who were part of an infection chain that included the participant. (See 12 for a fuller explanation of this.) Eligibility criteria included ability to answer the questionnaire; age ≥18 years; and being qualified for one of the project arms.

Potential seeds were recruited by referral from the Odessa Regional Laboratory Center of the Ministry of Health of Ukraine, the Odessa AIDS Center, or the Way Home (a collaborating community organization). They were classified as “recently infected seeds” if they were newly HIV‐diagnosed drug injectors or others who had LAg OD_n_≤1.5, with viral load >1000 copies/mL or had a documented confirmed negative HIV test within the prior six months. (LAg and other assays are described in more detail below.) Only three of the 24 recently infected seeds were classified as such due to having a documented negative HIV test within the last six months. For this paper, we defined intervention arm members as risk network members of recently infected seeds (but not the seeds themselves).

Comparison arm seeds were HIV‐positive participants recruited from the same referral sources as recently infected seeds, but who did not meet the criteria to be classified as recently infected (i.e. had LAg ODn>1.5 without any evidence of seroconversion in the last six months). Longer‐term infected seeds were matched to recent seeds for risk group, age (±5 years) and gender. The comparison arm consisted only of risk network members of these seeds.

#### 2.2.2 TRIP HIV assays

Blood samples were tested for HIV by New Vision Diagnostics Profitest Combo tests (Intec Products Inc.) and confirmed by re‐testing with Profitest. HIV‐positive specimens were tested for viral load using HIV‐1 Abbott RealTime^TM^ assay and were also tested for recent infection with the Limiting Antigen Avidity (LAg) assay (Sedia^TM^ Biosciences Corporation). The LAg assay is based on antibody avidity maturation and categorizes HIV infection as recent or long‐term based on a normalized optical density (ODn) cut‐off of 1.5 22, corresponding to a recency window period of 130 days 23, 24. All samples with OD_n_<0.4 were confirmed as HIV seropositive by re‐testing with either HIV‐1/2 Ag + Ab Ultra MBA 0416/5 assay (MedBioAkliance, Ukraine) or with Genscreen Ultra Ag/Ab, 6E0720 assay (France). Samples were considered to be recently infected if found HIV positive by either test, unless they had a viral load of ≤1000 copies/mL.

#### 2.2.3 TRIP questionnaire

The TRIP face‐to‐face interview included questions about socio‐demographic characteristics, sexual orientation, sexual and injection risk behaviours (including numbers of same‐ and opposite‐sex sex partners) in the last six months, and treatment history. It also asked participants to name risk network members in the following categories: people they injected drugs or had sex with in the past six months; people who injected or had sex in their presence in the past six months; and people who injected, used drugs or had sex with people with whom the participants had injected or had sex. Respondents also were asked about “venues” they usually visited to use drugs, to have sex or to meet new sex partners.

#### 2.2.4 TRIP network tracing

Risk network members (including participants recruited from “venues” that participants named) of recently infected and longer‐term infected seeds were recruited regardless of their infection status, as were the risk partners and venue members of these network members. In other words, network members were recruited for at least two “steps” from seeds. If a PwRI was found in networks of seeds (including those recruited through venues), the network members of this newly identified recently infected network member were recruited for two additional steps.

#### 2.2.5 TRIP dependent variable

The dependent variable in the analyses was the proportion of network members in each study arm who were PwRI. Network members were tested for HIV. If they were HIV positive, we carried out LAg tests and quantified plasma HIV‐RNA. Recently infected network members were defined as people with LAg OD_n_≤1.5 (and viral load >1000 copies/mL) since we had only self‐reports of network members’ testing history. Longer‐term infected network members were infected members of seeds’ networks who were not classified as recently infected.

#### 2.2.6 Incentives and benefits of participation in TRIP

TRIP participants received 50 hryvnia (approximately US$6 in 2013; US$2 in 2016) for baseline interviews and follow‐up interviews; 20 hryvnia (approximately US$2.50 in 2013; approximately $0.80 in 2016) for every named network member who brought in a referral coupon from the participant; and 10 hryvnia (approximately US$1.25 in 2013; approximately $0.40 in 2016) for every person recruited from a venue the participant named during the interview. The number of nominated network members and of venue members (whom our staff social worker recruited) was not limited.

Project staff educated affected participants and communities about recent/acute HIV infection, and about the importance of avoiding stigma. As discussed in other articles 25, 26, neither project staff nor participants in our project and a precursor pilot study in Ukraine reported any increases in stigma‐related problems, but participants did report significantly higher levels of experienced social support at follow‐up than at baseline. Participants were provided with standard counselling and were actively linked to care if appropriate.

### 2.3 IBBS methods

#### 2.3.1 IBBS overview and questionnaire

IBBS among people who inject drugs (PWID) in 2013 was a cross‐sectional Respondent‐Driven Sampling (RDS) study in 29 Ukrainian cities (including Odessa, for which N = 400) 27) designed to estimate the parameters of the HIV epidemic (rather than to locate PwRI for intervention). Trained experienced interviewers conducted face‐to‐face interviews using an adapted version of a previous IBBS questionnaire for PWID (2007 to 2011). Questions included socio‐demographic characteristics, sexual and injection behaviour in the last 12 months, including whether men had sex with men in the last 12 months, and previous HIV‐testing experience. Experienced medical workers conducted HIV rapid tests after the interview for all participants.

#### 2.3.2 IBBS eligibility and recruitment

Participants were enrolled in IBBS after preliminary screening based on the following criteria: that they had injected drugs within the last 30 days, were at least 14 years old, currently resided in Odessa and had not participated in any other surveys within the last six months. A medical worker checked veins for signs of punctures. Only PWID with visible punctures were allowed to participate.

Seeds in Odessa, as well as in other IBBS cities, were people who met the above criteria. They were selected to make sure that the seeds selected from each city had unknown or negative HIV status and varied in terms of age and other important characteristics. Specifically, they were selected such that they included at least one 14 to 19 years old, at least one woman, and at least one with each of the following characteristics: less than two years of injection experience; exclusive stimulants user; exclusive opioid user; stimulant and opioid mixed user; NGO client; and non‐NGO client. Odessa IBBS had four seeds. Two were recruited from The Way Home (the NGO where TRIP also recruited seeds), one was recruited from another NGO in another part of the city, and one was recruited independently of NGOs.

Seeds were given three coupons to give to other PWID who could then take part in the study and receive compensation, as described below.

#### 2.3.3 IBBS assays

All participants were tested for HIV using CITO TEST HIV‐1/2/0 rapid test kit. Dry blood spot specimens were collected from all participants who had HIV‐positive rapid test results and sent for laboratory testing at the United States Centers for Disease Control and Prevention (CDC) by two third generation HIV diagnostic ELISAs to confirm the presence of HIV antibodies (Abbott ARCHITECT HIV Ag/Ab Combo (USA) and Bio‐Rad Genscreen Ag/Ab HIV Ultra (USA)). Samples that tested reactive on both ELISAs were confirmed for HIV seropositivity using Western blot (Inno‐lia HIV‐1/2 Score, Innogenetics, Belgium).

HIV‐positive specimens were tested by the Sedia LAg assay to determine if the infection had been recently acquired. Specimens with LAg ODn ≤1.5 were considered possible recent HIV‐1 infections. All specimens with ODn >1.5 were classified as long‐term infection.

All specimens with ODn ≤1.5 were tested for viral RNA using an adapted SOP for DBS on the Abbott m2000rt Real Time HIV Test. Similar to TRIP, specimens with VL ≥1000 copies/mL were considered to be confirmed recent HIV‐1 infection cases. Specimens with VL <1000 copies/mL were classified as long‐term infections.

#### 2.3.4 IBBS dependent variable

The dependent variable for IBBS was the proportion of participants who were PwRI. IBBS participants were defined as PwRI, longer‐term infected, or uninfected by the above assays.

#### 2.3.5 Incentives and benefits of participation in IBBS

IBBS participants received compensation for their participation with 30 hryvnia (US$4 in 2013), plus 20 hryvnia (US$2.50) for the recruitment of each secondary participant according to RDS methodology.

*Cost comparison analyses* used simplified ingredients‐based methods 28. Recruitment cost, staffing cost, and assay‐processing cost data were available through Alliance for Public Health administrative records. These were compared between TRIP and IBBS to calculate the costs for each project. These totals were divided by the numbers of PwRI located by each project to calculate their cost per PwRI located. Both IBBS and TRIP involved large research components whose costs were excluded for these comparisons since they were not part of the intervention. Costs for IBBS were calculated on the basis of the exchange rate in 2013 when IBBS was conducted. At that time, the hryvnia (UAH) was valued at US$0.125. Costs for TRIP were estimated both using the same exchange rate and also on the basis of the exchange rate (<US$0.04 per UAH) at the time TRIP ended data collection (2016).

##### Analyses

Cross‐tabulations and frequencies were calculated using SPSS version 21. Odds ratios were used as a measure of association in cross‐tabulations; the statistical significance of these odds ratios was assessed using both χ^2^ and Fisher's Exact Test, which was necessary in most cases since there was only one recently infected participant in IBBS. In other words, we used the more conservative Fisher's exact test to test the null hypothesis that each sample had equal proportions of recently infected persons as a way to deal with this issue of data sparseness 29. Since the data are not based on probability samples, these *p*‐values should be viewed as heuristic estimates. Furthermore, since there was only one recently infected participant in IBBS, this sparse data problem meant that we could not compute meaningful confidence intervals for the odds ratios comparing the yields of TRIP and IBBS. We thus used exact statistics to test the null hypothesis that each sample had equal proportions of recently infected persons. Since TRIP recruited non‐PWID, additional analyses were conducted to compare the PWID subset of TRIP with IBBS. The sparseness issue meant that we could not confidently compute adjusted odds ratios for comparisons of IBBS and PWID subsamples of TRIP, since regression coefficients would be too dependent on the value, for each variable, of the one recently‐infected IBBS sample member. We therefore could not test for confounding with personal characteristics of participants.

##### Human subjects

TRIP participants gave informed consent under protocols approved by the IRBs of the National Development and Research Institutes and the Medical Ethics Committee at Gromashevskii Institute of Epidemiology and Infectious Diseases. IBBS participants gave informed consent under protocols approved by Medical Ethics Committee at Gromashevskii Institute of Epidemiology and Infectious Diseases and the Sociology Association of Ukraine. The recent infection component of IBBS was approved by the Medical Ethics Committee at Gromashevskii Institute of Epidemiology and Infectious Diseases. Prior to study enrolment, both seeds and secondary respondents were provided with comprehensive information about the study and signed a consent form.

## 3 Results

Participants’ characteristics are presented in Table 1, along with statistical comparisons of IBBS participants to TRIP's subsample of participants who were PWID. Both groups were about 83% male, and both had a median age of 35. IBBS participants (all PWID) had a longer mean duration of injection drug use (16.5 vs. 14.7 years). Compared to the IBBS sample, the TRIP PWID subsample had considerably higher proportions of participants who were homeless, who were in drug or alcohol treatment, and who were unemployed or unable to work. Participants in the two arms of the TRIP sample were generally similar. One difference was that the TRIP network members of PwRI were more likely not to be in drug or alcohol treatment than were the TRIP network members of longer‐term infected seeds (OR = 3.04; 95% CI 1.89, 4.88). By self‐report, 18 (1.4%) of the male members of TRIP networks were MSM in the last six months, and no IBBS participants were MSM in the last year. None of these MSM had recently been infected. Twelve (67%) of the MSM in the TRIP networks were HIV‐positive.

**Table 1:** Characteristics of participants in TRIP networks and IBBS in Odessa

	TRIP networks total	TRIP networks PWID only	IBBS (unweighted)	TRIP PWID versus IBBS (difference)	TRIP networks of PwRI	TRIP networks of long‐term infected
Total	1252	551	400		735	517
Males	993 (79.3%)	471 (85.5%)	328 (82.0%)	χ^2^ = 2.09; *p* = 0.148	579 (78.8%)	414 (80.1%)
Median age in years (IQR)	34 (27 to 41)	35 (29 to 41)	35 (29 to 42)	t = 1.08; *p* = 0.280	34 (28 to 41)	34 (27 to 41)
Education–at least high school (11 years) completed	980 (78.3%)	434 (78.8%)	315 (78.8%)	χ^2^ = 0.03; *p* = 0.867	558 (75.9%)	422 (81.6%)
Homeless	168 (13.4%)	54 (9.8%)	1 (0.3%)	χ^2^ = 39.10; *p* < 0.0005	110 (15.0%)	58 (11.2%)
PWID^a^ (injecting over the last six months)	551 (44.0%)	551 (100%)	400^a^ (100%)	N/A	303 (41.5%)	248 (48.0%)
Duration of injection in years	Not applicable, see next column	14.7 (7 to 21.25)	16.5 (10 to 22)	t = 2.79; *p* = 0.005	15 (8 to 22)	15 (7 to 21)
On drug/alcohol treatment at enrollment	102 (8.1%)	54 (9.8%)	9 (2.3%)	χ^2^ = 21.36; *p* < 0.0005	35 (4.8%)	67 (13.0%)
Unemployed/unable to work	496 (39.6%)	256 (46.5%)	89 (22.3%)	χ^2^ = 59.11; *p* < 0.0005	264 (35.9%)	232 (44.9%)
Sex workers	4 (0.3%)	2 (0.4%)	0%	χ^2^ = 1.46; *p* = 0.228	3 (0.4%)	1 (0.2%)
Male sex workers (% of males)	1 (0.1%)	1 (0.2%)	0%		1 (0.2%)	0 (0.0%)
Female sex workers (% of females)	3 (1.2%)	1 (1.3%)	0%		2 (1.3%)	1 (1.0%)
Engaged in male/male sex (% of men) in last six months (TRIP) or last 12 months (IBBS)	18 (1.4%)	2 (0.4%)	0%	χ^2^ = 1.46; *p* = 0.228	3 (0.4%)	15 (2.9%)

a IBBS participants were all PWID.

Of those recruited in recently infected seeds’ risk networks, 85.3% were recruited from venues. In the longer‐term infected seeds’ risk networks, by contrast, 71.7% were recruited from venues. The 28.3% who were recruited by normal network sampling procedures in the longer‐term HIV‐positives’ risk networks was almost twice (ratio = 1.92) the percentage (14.7%) for the PwRI seeds’ networks. (By way of comparison, in the Athens TRIP project, only 6.5% of the PwRI seeds’ risk networks were recruited from venues, as were 5.9% of the longer‐term seeds’ risk networks.)

About 2% of the network members in each TRIP arm were PwRI. The odds ratios’ confidence intervals overlapped unity. (Table 2).

**Table 2:** Percent of recently HIV infected people in the TRIP network‐recruited participants and their Arms as compared with IBBS2013 surveillance programme

	N	N recently infected in networks	% Recently infected in networks
Network of recent seeds	735	13	1.8%
Networks of long‐term HIV‐positive seeds	517	11	2.1%
Total networks (adding Recents’ networks together with long‐term positives’ networks)	1252	24	**1.9%**
IBBS 2013	400	1	**0.25%**
Comparison of network of recent seeds to network of long term HIV‐positive seeds	OR 0.83 CI 0.37, 1.86 χ^2^ = 0.21, *p* = 0.648; Fisher's Exact Test *p* = 0.679		
Comparison of network of recent seeds to IBBS Odessa	OR 7.18 χ^2^ = 4.90, *p* = 0.027; Fisher's Exact Test *p* = 0.025		
Comparison of network of longer‐term HIV‐positive seeds to IBBS Odessa	OR 8.67 χ^2^ = 6.16, *p* = 0.013; Fisher's Exact Test *p* = 0.016		
Comparison of both networks to IBBS Odessa	**OR 7.80** χ^2^ = 5.65, *p* = 0.017; Fisher's Exact Test *p* = 0.016		

The 2% proportion of PwRI in TRIP networks was higher than the 0.25% proportion in IBBS. The odds ratios for comparisons of the TRIP PwRI seeds’ networks, the TRIP longer‐term infected seeds’ networks, and the combination of the two arms, as compared to IBBS, were all greater than 8.0, and the 95% confidence intervals for each odds ratio remained above 1.5.

Among PWID, approximately 3% of network members from each arm were PwRI. The odds ratios for the proportion of PwRI in each TRIP arm as compared to the IBBS sample, and for the network members from both TRIP arms combined as compared to the IBBS sample, were all 9.0 or more (see Table 3). Among the non‐PWID network members from TRIP (Table 4), the proportion recently infected in each arm was approximately 1.4%. No comparisons can be made to IBBS, since all IBBS participants were PWID.

**Table 3:** Percent of recently HIV‐infected *people who inject drugs* in the TRIP network‐recruited participants and their Arms and comparison with data in Table 2 for IBBS2013 surveillance programme

PWID only	N	N recently infected in networks	Percent recently infected in networks
Network of recent seeds	303	7	2.3%
Networks of long‐term positive seeds	248	8	3.2%
Total networks (adding recents’ networks together with long‐term positives’ networks)	551	15	**2.7%**
IBBS 2013	400	1	**0.25%**

Comparison of network of recent seeds to network of long term HIV‐positive seeds	OR 0.71 CI 0.25, 1.98 χ^2^ = 0.43, *p* = 0.511; Fisher's Exact Test *p* = 0.602
Comparison of networks of recent seeds to IBBS Odessa	OR 9.44 χ^2^ = 6.51, *p* = 0.011; Fisher's Exact Test *p* = 0.024
Comparison of network of networks of longer‐term HIV‐positive seeds to IBBS Odessa	OR 13.30 χ^2^ = 9.90, *p* = 0.002; Fisher's Exact Test *p* = 0.003
Comparison of both TRIP networks to IBBS Odessa	OR 11.17 χ^2^ = 8.56, *p* = 0.003; Fisher's Exact Test *p* = 0.003

**Table 4:** Percent of recently HIV‐infected people who *do not* inject drugs in the TRIP network‐recruited participants and their Arms^a^

NON‐PWID Only	ALL	N recently infected in networks	Percent recently infected in networks
Network of recent seeds	427^b^	6	1.4%
Networks of long‐term HIV‐positive seeds	267^b^	3	1.1%
Total networks (adding Recents’ networks together with long‐term positives’ networks)	694^b^	9	1.3%
OR (CIs) for comparison of arms		OR 1.25; CI 0.31, 5.06 χ^2^ = 0.10; Fisher's Exact Test *p* = 1.00	

^a^Since IBBS2013 only studied people who inject drugs, this table only includes TRIP participants; ^b^A total of seven participants were missing data on drug injection (five in the networks of recent seeds; two in the networks of long‐term HIV‐positive seeds).

Cost comparison analyses (Table 5) found that it cost TRIP approximately $1800 to locate each recently infected participant using the exchange rate operative during most of the TRIP data collection period, or approximately $4500 using the exchange rate in 2013. (The Ukrainian currency was sharply devalued following the Maidan insurrection that ousted the President early in 2014, the Russian occupation of Crimea, and the beginning of civil war in eastern Ukraine.) The cost to locate a recently infected participant in IBBS in 2013 was $11,924.

**Table 5:** Cost comparison^a^

Items	Comments	Cost, US$ using exchange rate of 25.59 UAH per US dollar	Quantity	Total cost, $
a. TRIP November 2013 to March 2016				
Staff costs storefront				
Interviewer	53 interviews per month; 33 hours per week per person; two persons	10.55	1452	15320.05
Social worker per month	25 hours per week	136.77	28	3829.62
Medical staff				
Nurse per month	4 per day	117.23	28	3282.53
Recruitment costs				
Interview		1.95	1452	2837.05
Contact		0.78	1452	1134.82
Place		0.39		
Test procurement				
Rapid test	For detection	1.00	1452	1452.00
Rapid test	In Lab for HIV positive	1.00	356	356.00
LAg	Per test	10.89	356	3878.30
Viral load	Per test	22.26	356	7926.38
Lab labour				
LAg	Per test conducted	3.13	356	1112.93
Viral load	Per test conducted	5.86	356	2086.75
Total cost				43,216.43
Number of people tested			**1452**	
Number of HIV positive			**356**	
Number of recently infected participants detected			**24**	
**Cost per recently infected participant detected if exchange rate is valued at time TRIP stopped collecting data**				1800.69
**Cost per recently infected participant detected if exchange rate were 8 UAH per dollar**				**4512.82**

Items	Comments	USD	Quantity	Total cost, $
b. IBBS (38 days of actual data collection)				
Site staff				
Interviewer		4.25	400	1700.00
Coupon manager		410.25	1	410.25
Regional supervision		512.88	1	512.88
Medical staff				
Nurse	Per test	1.71	400	685.00
Doctor	Pre‐ and post‐test counseling	1.71	400	685.00
Nurse	Per dried blood spot	1.71	108	184.95
Supervision of biological aspects		307.75	1	307.75
Recruitment cost				
Interview		4.25	400	1700.00
Recruiting		2.56	400	1025.00
Test procurement				
Rapid test	For detection	1.00	400	400.00
Dried blood spot	For all HIV positive	1	108.00	108
LAg	Per test	10.89	108	1176.12
Viral load	Per test	22.26	108	2404.08
Training the staff	Per training	357.00	1	312.50
Site rent		312.50	1	312.50
Total cost per period				11,924.03
Number of people tested			**400**	
Number of HIV positive			**108**	
Number of recently infected participants detected			**1**	
**Cost per recently infected participant detected**				**11,924.03**

a Assumptions: UAH is the Ukrainian hryvnia. The analysis uses an exchange rate of 25.59 UAH to the US dollar for TRIP costs except where otherwise indicated. For IBBS, the analysis uses the rate of 8 UAH to the dollar, which was the rate in 2013 when the data were collected. The IBBS costs for the LAg and Viral load were set equal in US dollars to those for TRIP. (CDC conducted these tests for IBBS for no charge as part of its research; but to compare programme costs, we need to treat these tests as part of the programme.)

## 4 Discussion

Both arms of TRIP located and recruited a considerably larger proportion of PwRI than did IBBS. This was also true when comparisons were restricted to PWID. Furthermore, cost comparisons indicate that TRIP techniques locate PwRI less expensively than does IBBS. These findings support the conclusions of a TRIP‐related study in Athens, Greece 13, which concluded that “efforts to seek, test, and treat PwRI can be accelerated using strategic, network‐based approaches.” The results in this paper show that, in Odessa, the risk network recruitment approach used in TRIP, together with recruiting seeds who were HIV positive, located more PwRI (at less expense) than a standard RDS epidemiologic study.

Contrary to our expectations, the two arms of TRIP recruited similar proportions of PwRI in Odessa. In Athens TRIP, the networks of PwRI contained higher proportions of PwRI than did the networks of longer‐term HIV‐positives 13. These different results may well be due to the different percentages in the two cities of risk network members who were recruited from venues as opposed to from among named risk network members. It seems likely that many of these venue recruits did not engage often, if at all, in risk behaviours with those who nominated their venues as places from which to recruit, so their probability of being recently infected would be likely to have a weaker relationship to the infection status of the seed or network member from whose venue they were recruited. However, our sample size was unfortunately too small to let us test for a possible effect on PwRI recruitment rate of the interaction between recruitment type (venue vs. direct risk partner recruitment) and TRIP arm (networks of PwRI seeds vs. longer‐term HIV‐positive seeds).

This paper is subject to a number of limitations. First, networks may have been under‐recruited in TRIP due to under‐reporting of risk partners’ names or of venues that participants frequented, or due to our being able to recruit only some of those named (since some participants were unwilling or unable to recruit some of their named network members). Second, in TRIP, but not in IBBS, seronegative network members could be re‐interviewed and re‐tested after six months if they were recruited as a network member again – which might have slightly increased the number of PwRI recruited by TRIP. Third, both TRIP and IBBS relied on monetary incentives to increase participation. This led to some participants incorrectly taking part more than once. Fourth, IBBS excluded seeds who were already aware (and self‐reported) that they were HIV positive, whereas TRIP sought out and tested seeds who were already tested and confirmed to be HIV positive. Although this might have biased TRIP networks to include more HIV‐positives, it is unclear whether this difference would bias TRIP or IBBS to recruit more *recently infected* network members. This is because HIV negatives are the only people who can get infected and thus become recently infected, so IBBS seeds (who self‐reported that they were HIV negative and were therefore likely to be either uninfected or HIV‐positive unaware) might be more likely to be in networks with recently infected people. Fifth, the seeds for both arms of TRIP were clients of the same community service organization, so their networks often overlapped. This may have contributed to making their yields of PwRI similar. Sixth, TRIP recruitment occurred after IBBS recruitment, with most TRIP recruitment taking part after the beginning of widespread protests in late 2013 and the subsequent governmental changes, Russian take‐over of Crimea, insurgency in eastern Ukraine, and ensuing large‐scale migration of refugees. If these “Big Events” and their corresponding social and structural instability increased HIV incidence, this could have contributed to a portion of the measured TRIP/IBBS differences 30, 31. Seventh, the fact that only one IBBS participant was recently infected created a problem of sparseness, which means that the accuracy of the magnitude of the odds ratios is limited. This same problem prevented adjusted analyses to control for confounding covariates. As Greenland et al. 29 suggest, we analysed the bivariate associations between yields for IBBS and TRIP both for the whole TRIP sample and for the subsample of people who inject drugs using the (conservative) Fisher's Exact Test. In both cases, the comparisons had significance levels below 0.05. A final limitation is that TRIP was designed to recruit PwRI for intervention purposes, whereas IBBS had an epidemiologic focus, which might have influenced their relative yields of PwRI.

Past research on injectors’ networks in Athens by Nikolopoulos et al. 13 and research by Green et al. in San Diego 32 showed that partner services that traced the sexual contacts of the recently infected are effective at finding other PwRI. Taken together with this paper, this pattern of results suggests that risk network (although perhaps not venue tracing) methods starting with either recently‐ or longer‐term HIV‐infected people are effective ways to locate PwRI. As discussed above, PwRI are a strategically important target group for treatment as prevention. A recent review of HIV‐related network research and interventions suggests ways in which such interventions and research can be effectively conducted 33.

Further research is needed to confirm these results and to understand what epidemiologic and sociocultural circumstances and/or intervention techniques affect the relative proportions of PwRI found in the networks of recently infected seeds and those of seeds with longer‐term infection. Future research should also make direct statistical comparisons between venue‐based recruitment and recruitment of direct risk partners. Research might also develop easier‐to‐implement ways to recruit risk network members and to search for PwRI.

## 5 Conclusions

Locating people soon after they become infected is an important goal both for patient care and for HIV prevention. The present findings suggest that network approaches may cost‐effectively identify PwRI at higher rates than does RDS. Network‐tracing interventions that start with people who test HIV positive are a cost‐effective way to locate PwRI and should be widely implemented.

## Competing interests

None to declare.

## Authors’ contributions

LDW Together with SRF, did most of the writing of the paper; helped conceptualize this paper; conducted most of the statistical analyses; contributed to TRIP field operations; helped resolve complex data issues; and approved the final version. AK Managed day to day TRIP field operations during much of the project; organized and maintained complex data files; helped conceptualize this paper; helped resolve complex data issues; contributed to the analyses; helped write the paper; and approved the final version. PS Helped write the funding proposal; oversaw and contributed to all TRIP field operations; helped conceptualize this paper; conducted the analyses of costs; helped write the paper; and approved the final version. YS Conducted analyses of the IBBS and Outreach Testing data; helped write the paper; and approved the final version. GKN Helped conceptualize this paper; helped design the analysis plan; helped write the paper; and approved the final version. BS Helped conceptualize this paper; helped write the paper; and approved the final version. EM Helped conceptualize this paper; helped write the paper; and approved the final version. JS Helped write the proposal; helped conceptualize this paper; helped write the paper; and approved the final version. TVY Helped write the funding proposal; managed day to day field operations during much of the project start‐up; set up and organized complex data files; helped write the paper; and approved the final version. YTD Trained and oversaw Odessa laboratory staff on LAg test; conducted LAg analyses at CDC for IBBS; helped write the paper; and approved the final version. SC, VC. LK conducted the operations of the Odessa Regional Laboratory Center of the Ministry of Health of Ukraine which conducted the LAg analyses and provided other information that help determine which participants had recently been infected; approved the paper. SRF Overall project principal investigator; oversaw all field operations in some detail; wrote funding proposal with help from PS, JS and TVY; led in conceptualizing and directing this paper; conducted some analyses; helped write the paper; approved the final version.
